# Molecular ground-state dissociation in the condensed phase employing plasmonic field enhancement of chirped mid-infrared pulses

**DOI:** 10.1038/s41467-019-11902-6

**Published:** 2019-08-29

**Authors:** Ikki Morichika, Kei Murata, Atsunori Sakurai, Kazuyuki Ishii, Satoshi Ashihara

**Affiliations:** Institute of Industrial Science, The University of Tokyo, 4-6-1, Komaba, Meguro-ku, Tokyo 153-8505 Japan

**Keywords:** Chemical physics, Nanophotonics and plasmonics, Ultrafast photonics

## Abstract

Selective bond cleavage via vibrational excitation is the key to active control over molecular reactions. Despite its great potential, the practical implementation in condensed phases have been hampered to date by poor excitation efficiency due to fast vibrational relaxation. Here we demonstrate vibrationally mediated, condensed-phase molecular dissociation by employing intense plasmonic near-fields of temporally-shaped mid-infrared (mid-IR) pulses. Both down-chirping and substantial field enhancement contribute to efficient ladder climbing of the carbonyl stretch vibration of W(CO)_6_ in *n*-hexane solution and to the resulting CO dissociation. We observe an absorption band emerging with laser irradiation at the excitation beam area, which indicates that the dissociation is followed by adsorption onto metal surfaces. This successful demonstration proves that the combination of ultrafast optics and nano-plasmonics in the mid-IR range is useful for mode-selective vibrational ladder climbing, paving the way toward controlled ground-state chemistry.

## Abstract (web summary)

There is a growing interest in controlling the chemical process at molecular level. Here the authors show the mid-IR chirped pulse driven ground-state dissociation of condensed phase tungsten hexacarbonyl using efficient vibrational ladder climbing process by employing plasmonic near-field enhancement.

## Introduction

Active control of chemical reactions on a molecular level, that is, selective breaking or making of chemical bonds, is one of the Holy Grails in physical chemistry^1^. Strong excitation of key reagent vibrations is a potentially powerful strategy for selective reaction control^2^, as has been validated by theoretical studies^3–6^. Vibrational motions may be activated through electronic excitation, but this scheme is restricted to modes accessible by Franck–Condon transitions and excited-state conical intersections^7^. In contrast, vibrational excitation via infrared (IR)/Raman processes at electronic ground states can be a more versatile technique due to its much broader accessibility to the potential energy surface. Compared with electronic excitation with ultraviolet/visible pulses, which causes various unwanted reactions, IR vibrational excitation is more advantageous for selectivity in reaction control.

Mid-IR ultrafast laser technologies have enabled mode-selective, multi-quantum vibrational excitation, and have opened a way to control molecular reactions at electronic ground states. Molecular dissociation mediated by vibrational ladder climbing (VLC) has been successfully achieved for gas-phase molecules^8–11^. Similar VLC has been performed on liquid-phase molecules as well^12–16^. Down-chirping proved useful for efficient VLC^10,12,14^, and arbitrary pulse-shaping was exploited for selective population of the excited vibrational levels^15^. These works have stimulated theoretical studies on the relevant topics^17–19^. However, attempts to observe ground-state dissociation in liquid phases^12–16,20^ have been unsuccessful to date, owing to the following reasons: collisions with solvent molecules accelerate vibrational relaxation to disturb deposition of sufficient energy on a specific mode, which would otherwise flow into the reaction coordinate, and dissociated species recombine on a short (~microsecond) timescale.

We may resolve these issues to open an avenue toward controlled ground-state chemistry by combining ultrafast laser technology with plasmonics in the mid-IR range. Plasmonics takes advantage of coupling of light to metal electrons, enabling subwavelength localization and strong field enhancements. Therefore, surface-plasmon excitation substantially enhances light–matter interactions. Such enhancements in the mid-IR range have been utilized in surface-enhanced infrared absorption spectrosopy^21–27^. Recently, surface plasmons have been successfully applied to nonlinear vibrational spectroscopy^28–32^, where third-order nonlinear signals were amplified by the near-field enhancement of mid-IR pulses. Here, it is important to note that higher-order nonlinear processes are more significantly enhanced because of their higher-order dependence on field strengths^33,34^. This fact suggests that plasmonic field enhancement may play a key role in accelerating VLC to enable sufficient energy deposition on a timescale faster than vibrational relaxation. Recently, surface-enhanced VLC has been demonstrated for a para-mercapto-benzonitrile adsorbed onto a rough metal surface but without any signs of reaction^35^.

**Fig. 1:**
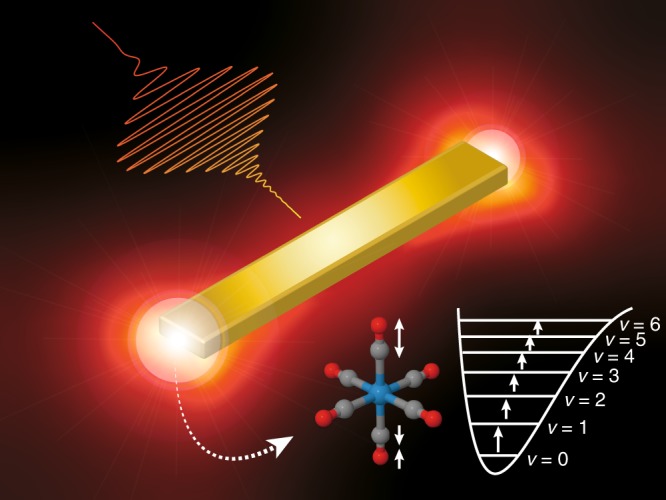
Schematic view of the antenna-enhanced vibrational ladder climbing. The chirped mid-IR pulse, enhanced with plasmon excitation of the gold nanoantenna, strongly excites the carbonyl stretch vibration of W(CO)_6_ molecules

In this paper, we realize the ground-state dissociation of condensed-phase molecules by employing chirped mid-IR pulses, substantially enhanced on a nanometer scale by plasmon excitation. In our reflection pump-probe experiments, mid-IR pump pulses are locally enhanced with plasmon excitation of gold nanoantennas to strongly drive VLC in the *T*_1*u*_ CO-stretching mode of W(CO)_6_ in *n*-hexane solution (Fig. 1). The resulting vibrational population distribution is sensitively probed as transient reflectance change, since population distribution is printed onto the reflectance spectrum of gold nanoantennas via antenna-molecule coupling. Both down-chirping and near-field enhancements contribute to increasing the climbing efficiency and allow the observation of vibrational population up to *v* = 6, whose energy is close to the CO-dissociation energy. Furthermore, we observe an absorption band emerging with mid-IR laser irradiation at the excitation beam area. These results show that the carbonyl compounds lose one CO ligand upon VLC, and the decarbonylated species are adsorbed onto the metal surfaces. The demonstrated scheme, where appropriately shaped mid-IR pulses are substantially enhanced in the vicinity of the metal surfaces, proved useful for mode-selective vibrational ladder climbing. This approach paves the way toward controlled ground-state chemistry in condensed phases, along with recent progress in other relevant topics^36–40^.

## Results

### W(CO)_6_/*n*-hexane solution on gold nanoantenna arrays

**Fig. 2:**
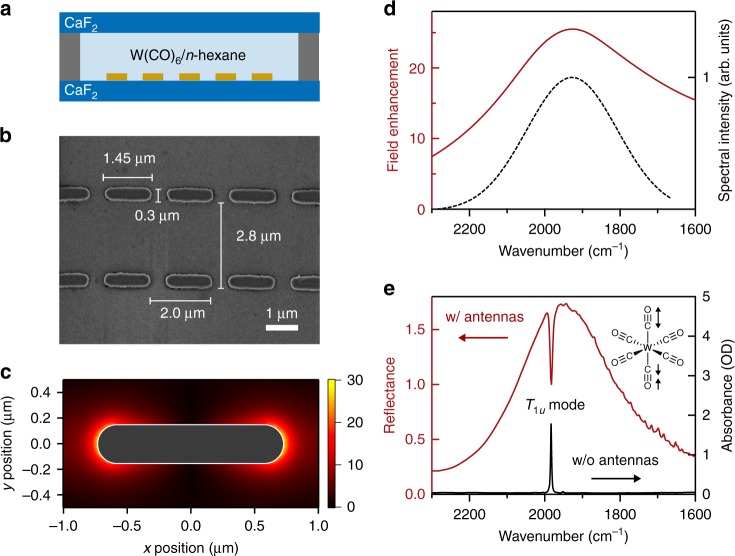
W(CO)_6_/*n*-hexane solution on gold nanoantenna arrays. **a** A schematic of the sample comprising two CaF_2_ windows separated by a Teflon spacer, and filled with W(CO)_6_/*n*-hexane solution. **b** The SEM image of the gold nanoantenna arrays. Each nanoantenna is 1.45-μm long, 0.3-μm wide, and 0.1-μm high. The size of the array unit cell is 2.0 μm × 2.8 μm. **c** Spatial distribution of the near-field enhancement factor simulated by the FDTD method. Dimensions of the nanoantenna are illustrated by a white line. **d** The simulated near-field enhancement factor in the vicinity of the nanoantenna ends (red) and the measured mid-IR laser pulse spectrum (black). **e** The measured reflectance for the nanoantenna arrays immersed in 20 mM W(CO)_6_/*n*-hexane solution (red) and the absorption spectrum for 3 mM W(CO)_6_/*n*-hexane solution (black)

Our sample is W(CO)_6_/*n*-hexane solution held between two CaF_2_ windows separated by a 25-μm Teflon spacer (Fig. 2a). The target mode is the *T*_1*u*_ CO-stretching mode of W(CO)_6_, which exhibits an absorption at a center frequency of 1983 cm^−1^ with a fwhm linewidth of 3 cm^−1^. For one of the two CaF_2_ windows, the rod-shaped gold nanoantennas are patterned into 2D arrays of rectangular lattice (see Methods for details). A scanning electron microscopy (SEM) image is shown in Fig. 2b. Figures 2c, d display the near-field distribution and the near-field enhancement spectrum of the nanoantenna arrays, respectively, simulated by the finite-difference time-domain (FDTD) method (see Supplementary Note 1 for details of the simulations). Here, we see that the nanoantennas exhibit localized surface-plasmon resonance at approximately the same frequency as the *T*_1*u*_ mode, with near-field enhancements in the vicinity of the nanoantenna ends.

### Antenna-molecule near-field coupling

The reflectance spectrum for the nanoantenna arrays immersed in 20 mM W(CO)_6_/*n*-hexane solution is shown in Fig. 2e. The broad peak of the antenna resonance is observed to be strongly modified at the resonance frequency of the *T*_1*u*_ mode with spectral features characteristic to Fano resonance^24,25^. This modification is caused by coupling to the molecular vibration (*T*_1*u*_ mode) in the following manner^27^. The incident field excites the plasmon mode, which in turn excites the molecular vibration via the near-field coupling. Next, the molecular vibrational excitation acts back to excite the plasmon mode. In this manner, coherent dipole excitation is continuously exchanged between the nanoantenna and the molecules until it is damped. The modification in the antenna reflectance results from such interference among direct/indirect plasmon excitations (see Supplementary Note 3 for details).

### Transient spectroscopy of uncoupled W(CO)_6_

We perform mid-IR pump-probe spectroscopy for probing of VLC in the *T*_1*u*_ mode (see Methods and Supplementary Fig. 1 for a detailed description of the experimental setup). We use mid-IR pulses with a temporal duration of 100 fs, a center frequency of 1930 cm^−1^, and a repetition rate of 1 kHz. The fwhm bandwidth of 280 cm^−1^ provides spectral overlap with higher transitions of the anharmonic ladder. The linewidth of the antenna resonance is broad enough to cover the mid-IR pulse spectrum (Fig. 2d), which indicates that the enhanced near-field exactly follows the temporal waveform of the input pulse except for a *π*/2-shift of the carrier phase (see Supplementary Note 1).

**Fig. 3:**
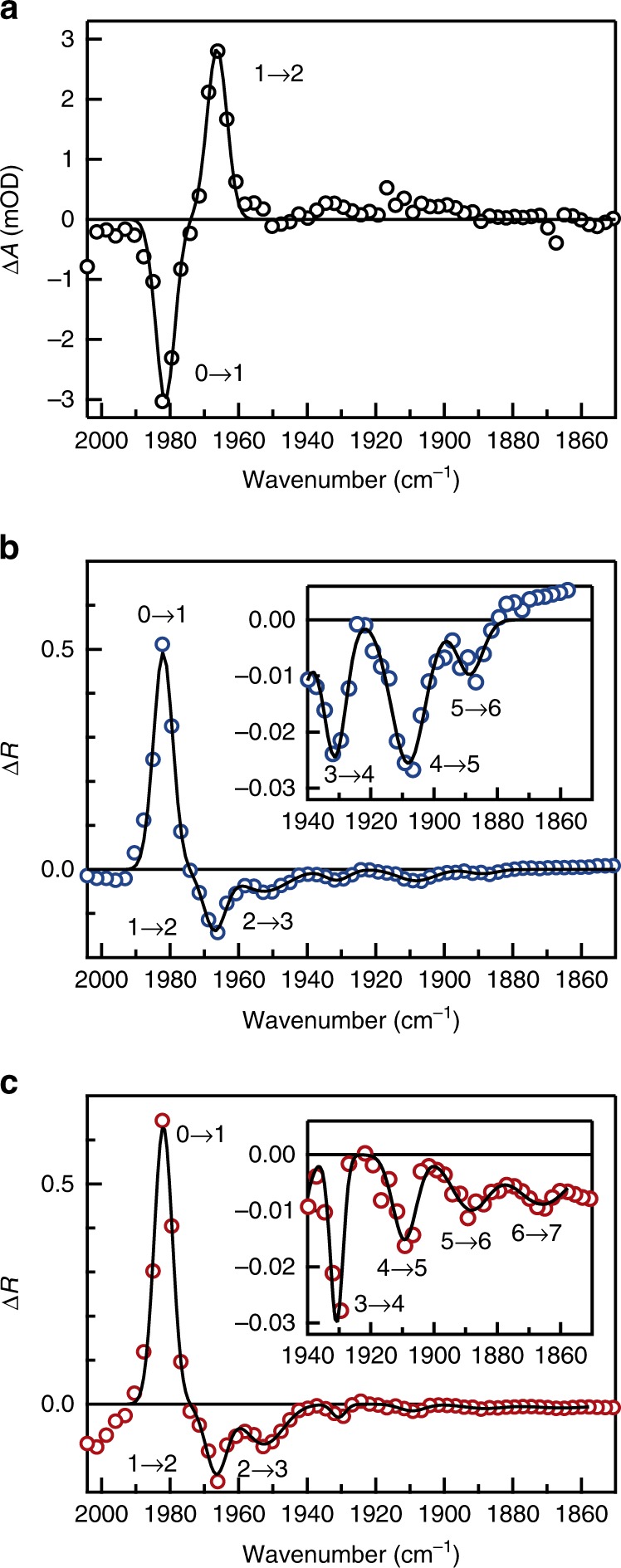
Transient spectra for uncoupled/coupled W(CO)_6_ molecules. **a** The transient absorbance change of uncoupled W(CO)_6_ at a 2-ps time delay after excitation with nearly-FTL pulses. **b**, **c** The transient reflectance change of coupled W(CO)_6_ at a 2-ps time delay after excitation: **b** with nearly-FTL pulses, and **c** with down-chirped pulses. Each black line represents a linear combination of multiple Gaussian functions, fitted to the measured absorbance/reflectance change

As a reference experiment, we measure the transient absorption of the uncoupled W(CO)_6_/*n*-hexane solution (3 mM) in a transmission geometry using nearly Fourier-transform limited (FTL) pump pulses with a fluence of 0.2 mJ cm^−2^. Here, we report on the signal in terms of the absorbance change, $\Delta {\it{A}} = - {\mathrm{log}}_{10}\left( {{\it{T}}/{\it{T}}_0} \right)$, where *T* and *T*_0_ denote the probe transmittance with and without pumping, respectively. Figure 3a shows the transient absorbance change at a 2-ps time delay, which is long enough to avoid the pump-probe coherent artifacts. After excitation, the uncoupled molecules exhibit a negative absorption feature at the fundamental *T*_1*u*_ mode frequency (1982 cm^−1^), attributed to the ground-state bleach (*v* = 0 → 1) and the stimulated emission (*v* = 1 → 0). They also exhibit a positive absorption feature at 1967 cm^−1^, attributed to the excited-state absorption (*v* = 1 → 2). These resonance frequencies are obtained by fitting the spectrum with a linear combination of Gaussian functions.

### Transient spectroscopy of antenna-coupled W(CO)_6_

We measure the transient reflectance of the antenna-coupled W(CO)_6_/*n*-hexane solution (20 mM), and report on the signal in terms of the reflectance change, $\Delta {\it{R}} = {\it{R}} - {\it{R}}_0$, where *R* and *R*_0_ denote the probe reflectance with and without pumping, respectively. Figure 3b shows the transient reflectance change at a 2-ps time delay, measured with the nearly-FTL pump pulses at a fluence of 0.2 mJ cm^−2^. After excitation, the antenna-coupled system exhibits a positive reflectance change at 1982 cm^−1^, attributed to the ground-state bleach (*v* = 0 → 1) and the stimulated emission (*v* = 1 → 0). They also exhibit negative reflectance changes, attributed to the excited-state absorptions: *v* = 1 → 2 at 1967 cm^−1^, *v* = 2 → 3 at 1953 cm^−1^, *v* = 3 → 4 at 1931 cm^−1^, *v* = 4 → 5 at 1908 cm^−1^, and *v* = 5 → 6 at 1889 cm^−1^. The sign inversion of the nonlinear signals, compared with the transmission measurements, is consistent with the fact that the peak feature observed for the uncoupled molecules in the linear absorbance manifests itself as the dip feature for the antenna-coupled molecules in the linear reflectance (Fig. 2e). These resonance frequencies, obtained by fitting the reflectance change with a linear combination of Gaussian functions, agree well with the energy levels expected for a Morse potential given by

1 $${\it{E}}_{\it{\upsilon }} = \hbar \omega _0\left[ {\left( {\upsilon + \frac{1}{2}} \right) - \chi _{\mathrm{e}}\left( {\upsilon + \frac{1}{2}} \right)^2} \right]$$

Here, the anharmonicity of *χ*_e_ ≈ 0.0049 and the corresponding anharmonic shift of 2*χ*_e_*ω*_0_ = 20 cm^−1^ (see Supplementary Fig. 3) are to be noted. These results are in line with those previously reported for uncoupled W(CO)_6_ molecules^13^. Therefore, the signals observed in our transient reflection spectroscopy are certainly attributed to the vibrational excitation of W(CO)_6_ molecules. It is noteworthy that vibrational excitation up to *v* = 5 is observed by employing plasmonic nanoantennas, despite the fact that the pump fluence is identical to that used for the uncoupled system shown in Fig. 3a.

Figure 3c shows the transient reflectance change at a 2-ps time delay measured with the down-chirped pump pulses (group-delay dispersion (GDD) of −24000 fs^2^) at a fluence of 0.2 mJ cm^−2^. Here, the temporal duration is stretched to ~1.3 ps (Supplementary Fig. 2e). In addition to the signals observed in Fig. 3b, we observe a signal at 1866 cm^−1^, attributed to the excited-state absorption of *v* = 6 → 7 transition. It indicates that the tungsten hexacarbonyl compounds are excited up to as high as *v* = 6. In the previous study^13^ on liquid-phase W(CO)_6_, vibrational excitation up to *v* = 5 was observed with nearly-FTL pump pulses at a fluence of 50 mJ cm^−2^. In this study, the irradiated pump fluence is as small as 0.2 mJ cm^−2^, but the pump fluence in the vicinity of nanoantennas is estimated to be 130 mJ cm^−2^, according to the simulated near-field enhancement of 25 as shown in Fig. 2c, d (comparison of VLC efficiency between experiments and density-matrix simulations suggests the effective near-field enhancement of ~14, as summarized in Supplementary Notes 2 and 3). Here, it is reasonably concluded that this large fluence as well as the down-chirping of the mid-IR pump pulses plays essential roles in realizing VLC up to the higher-lying states.

### Emergence of a new band

**Fig. 4:**
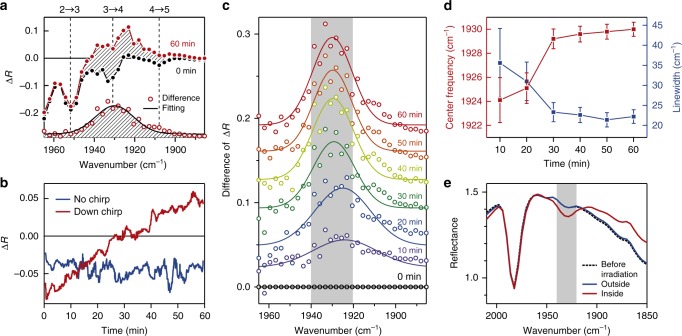
Cumulative evolution of an absorption band with pump irradiation. **a** Transient reflectance change at a 2-ps time delay for the antenna-coupled W(CO)_6_ before (black filled markers) and after (red filled markers) the 1-h irradiation with the down-chirped (GDD of −24,000 fs^2^) pump pulses at a fluence of 0.2 mJ cm^−2^. The red open markers represent the difference between the two reflectance changes, and the black dashed line represents a Gaussian function fitted to the data. **b** Time evolution of the reflectance change at 1930 cm^−1^ during the repeated irradiation of pump pulses: nearly-FTL (blue) and down-chirped (red). **c** Transient reflectance change at a 2-ps time delay for varied irradiation time, subtracted by the one for 0 min. Solid lines represent Gaussian functions fitted to the data (markers). **d** Center frequency and linewidth of the emerging band at different irradiation time, where the error is the standard deviation. **e** The linear reflectance spectrum measured after 1 h of the repeated irradiation for the area inside (red) and outside (blue) the irradiation beam spot. A black dashed line denotes the spectrum measured before the irradiation

We keep monitoring the pump-probe transient reflectance for the antenna-coupled molecules at a 2-ps time delay for an hour, while we irradiate the down-chirped pump pulses with GDD of −24,000 fs^2^ at a fluence of 0.2 mJ cm^−2^ with a repetition rate of 1 kHz. Figure 4a shows the transient reflectance change measured before and after an hour of pump irradiation. Note that the transient reflectance measured before the irradiation is essentially the same as the one shown in Fig. 3c. Surprisingly, the 1-h irradiation induces a change in the transient reflectance (also shown in Fig. 4a), i.e., there emerges an additional positive signal in the spectral range of 1910–1950 cm^−1^.

As shown in Fig. 4b, the transient reflectance change, Δ*R*, at 1930 cm^−1^ monotonically increases with irradiation time. This indicates that the positive signal, which is superposed onto the signal intrinsic to the original W(CO)_6_ molecules, grow cumulatively with the irradiation of down-chirped pump pulses. In contrast, Δ*R* does not evolve with time when we irradiate the sample with nearly-FTL pump pulses with the same fluence of 0.2 mJ cm^−2^ (the blue line in Fig. 4b). This striking contrast indicates the impact of down-chirping in emergence of the additional band.

Figure 4c displays the transient reflectance change at a 2-ps time delay for varied irradiation time, subtracted by the one for 0 min, which clearly shows a cumulative evolution of the positive signal. By the Gaussian-fitting analysis on each spectrum, we find that the center frequency is blue-shifted, and the linewidth is narrowed with irradiation time, as shown in Fig. 4d. After 1 h of the pump irradiation, the positive signal exhibits a center frequency of 1930 cm^−1^ and a fwhm linewidth of 22 cm^−1^.

After the 1-h irradiation with the down-chirped pump pulses, we measure the linear reflectance for the sample area of 200 μm × 200 μm inside/outside the irradiation beam spot (Pump: 200 μm, probe: 150 μm), shown in Fig. 4e. In addition to the original absorption dip of the *T*_1*u*_ mode at 1982 cm^−1^, an absorption dip is observed for the area inside the irradiation beam spot (not observed for the area outside the beam spot). Importantly, this dip exhibits the same center frequency and linewidth as the positive signal observed in the transient reflectance change with the pump irradiation. This coincidence indicates that the newly-appeared pump-probe signal shown in Fig. 4a–d originates from the ground-state bleach and the stimulated emission of the vibrational mode with its fundamental resonance at 1930 cm^−1^. Note that the corresponding excited-state absorption is not clearly observed at frequencies below 1930 cm^−1^. The reason is not clear, but it is possible that population of this emerging vibrational mode is distributed over many excited levels because of efficient ladder climbing and that the resonant excited-state absorption feature is smeared out because of the broad inhomogeneous linewidth.

### CO dissociation induced by chirp-pulsed near-fields

**Fig. 5:**
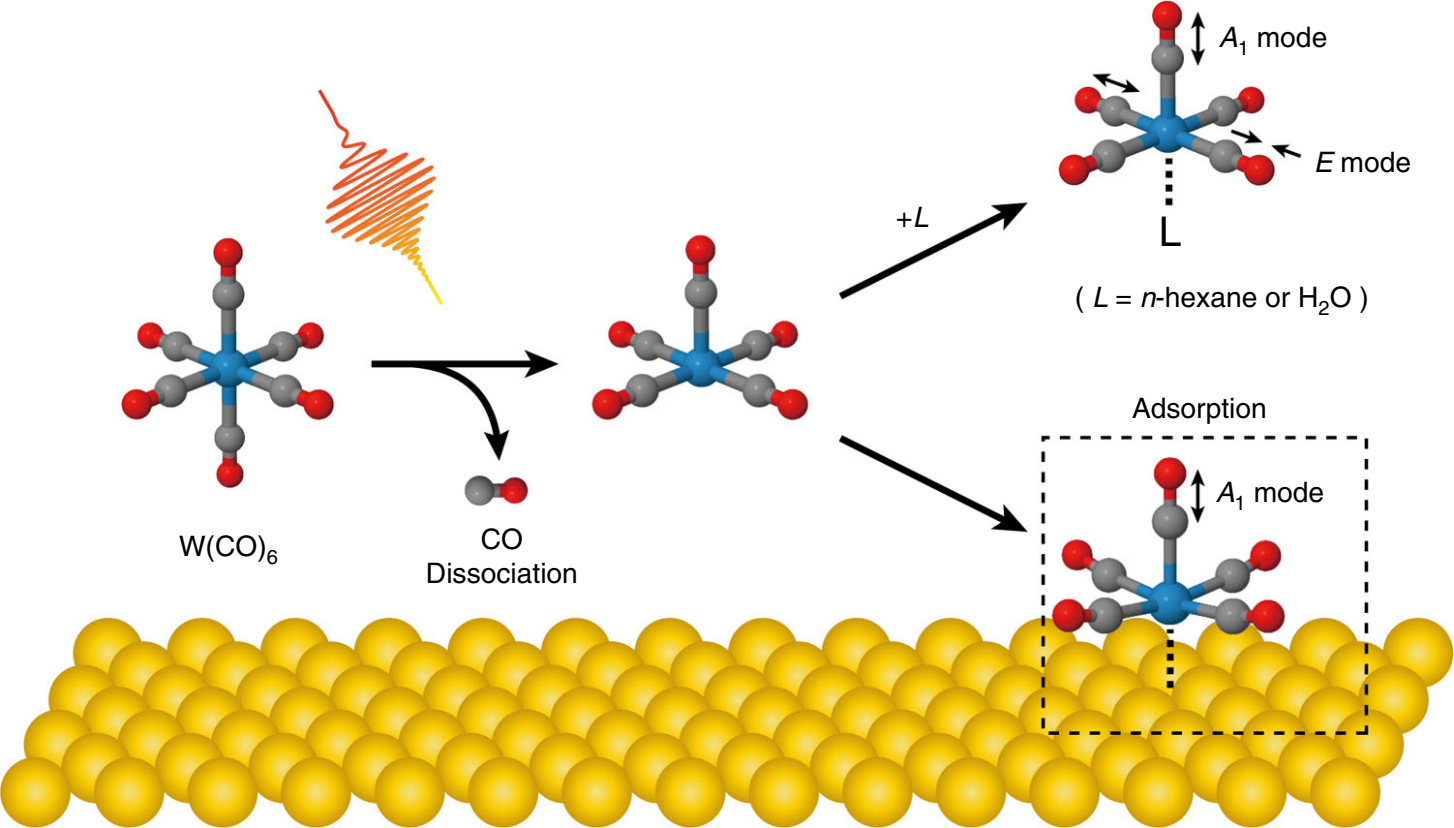
Proposed reaction schemes induced by antenna-enhanced vibrational ladder climbing. Upon multi-quantum vibrational excitation, the W(CO)_6_ molecule loses one CO ligand. The CO dissociation is followed by adsorption on the gold surface or coordination with the solvent molecule. The former species is sensitively detected, while the latter is undetectable in our antenna-enhanced spectroscopy

Upon the irradiation of down-chirped pump pulses, we observe VLC up to *v* = 6, whose energy is sufficient for the first bond dissociation^41,42^. This suggests that multi-photon excitation energy is channeled into a W-C bond via intramolecular mode-coupling, resulting in dissociation of a CO ligand (see Fig. 5). Here, we note that the energy corresponding to the *v* = 1 → 6 transition is sufficient for one CO dissociation, but insufficient for multiple CO dissociation^42^. Hence, W(CO)_6_ can lose only one CO ligand in each single shot process. Furthermore, because dissociated species recombine on a microsecond timescale in condensed phases^43^, shorter than the pump-pulse interval of 2 ms, multiple CO dissociation is much less probable than the single CO dissociation.

### Attribution of the final product

Spatial localization of this emerging band within the irradiation beam spot suggests that it originates from adsorbates, i.e., CO or W(CO)_5_ adsorbed on the gold surfaces. According to previous studies^44–50^, CO can be linearly adsorbed on top of a gold atom at the surfaces, and its stretching frequency varies in the range of 2000–2140 cm^−1^ depending on the surface condition. This frequency range is much higher than 1930 cm^−1^. Therefore, we can exclude the possibility of adsorbed CO as the origin of this absorption band. Note that we do not observe any sign of adsorbed CO on the linear reflectance spectrum at 2000–2140 cm^−1^ (see Supplementary Fig. 6).

According to the theoretical calculations based on density functional theory (DFT), W(CO)_5_ gets stabilized when adsorbed on the gold surface (see Supplementary Note 6 for details) and the fundamental transition frequency for *A*_1_ mode of adsorbed W(CO)_5_ is red-shifted by 45 cm^−1^ compared with that of the *T*_1*u*_ mode of W(CO)_6_. This frequency shift agrees well with the value of 53 cm^−1^ observed in our experiments. Here, we assign the absorption band observed at 1930 cm^−1^ to the *A*_1_ CO-stretching mode of W(CO)_5_ adsorbed on the gold surface (see Fig. 5). This assignment is in line with the electron-dose experiment for W(CO)_6_ adsorbed on the gold surface^51^, where the authors observed an absorption band originating from the decarbonylated species at ~55 cm^−1^ lower frequency than that of the parent molecule. Estimation of reaction efficiency is provided in Supplementary Note 5.

It should be noted that the W(CO)_5_ molecule adsorbed on the gold surface has a CO-stretching mode of *E*-symmetry as well. This *E* mode, however, is almost IR-inactive because it violates the surface selection rule, originating from the mirror-image dipole formation^52^. Furthermore, antenna-enhanced spectroscopy is much more sensitive to the *A*_1_ mode than the *E* mode because the transition dipole moment of the former is parallel to the surface normal as the plasmonic near-fields.

The gradual blue-shift of this absorption band with the pump irradiation (Fig. 4d) is explained by the dipole–dipole coupling among the adsorbate molecules^53,54^. In the present case, as the coverage with adsorbed molecules increases, the dipole–dipole interaction becomes stronger to induce a blue-shift. We can intuitively understand it by considering an array of interacting dipoles, where the restoring force acting on the oscillating dipoles is increased by the Coulomb force generated by the charge distributions of the neighboring dipoles.

It is also possible that the species dissociated with the pump pulse are coordinated by the solvent molecules. According to a previous study^43^, W(CO)_6_ in *n*-heptane solution loses one CO ligand upon UV excitation and forms W(CO)_5_L in several picoseconds. Subsequently, exchange of the solvent molecule with a water molecule occurs in a few hundred microseconds (water is a little impurity in the solvent). The same scenario can happen for the process after detachment of a CO ligand in our case of vibrational excitation, i.e. W(CO)_5_ forms W(CO)_5_(*n*-hexane) and then W(CO)_5_(H_2_O). The free CO and the W(CO)_5_(H_2_O) molecule, however, is undetectable in our antenna-enhanced spectroscopy because they quickly diffuse into bulk solution (see Supplementary Note 4).

## Discussions

We have demonstrated successful driving of ground-state dissociation of condensed-phase molecules by employing plasmonic near-fields of chirped mid-IR pulses. Both down-chirping and plasmonic field enhancement play essential roles in raising vibrational populations to higher-lying states and in realizing bond dissociation. The gold nanoantennas play an additional role in stabilizing the dissociated species prior to recombination. These key roles solve the long-standing issues of poor excitation efficiency and diffusional recombination that have hampered vibration-mediated dissociation of condensed-phase molecules. This successful demonstration proves that the combination of ultrafast optics and nano-plasmonics in the mid-IR range is useful for mode-selective, efficient vibrational excitation. The demonstrated scheme can develop into more powerful and versatile techniques by incorporating arbitrary pulse-shaping, well-designed plasmonic nanostructures, and functions of metal surfaces, paving the way toward controlled ground-state chemistry.

## Methods

### Sample fabrication

Gold nanoantenna 2D arrays of rectangular lattice are fabricated on a CaF_2_ substrate (20 mm in diameter, 1 mm in thickness). Electron beam resist (FEP-171) is spin-coated with a thickness of 300 nm, exposed by electron beam, and then developed. Subsequently, a 5-nm-thick Cr-adhesion layer followed by a 100-nm-thick Au layer are thermally evaporated and lifted-off by acetone.

### Spectroscopic measurements

Linear reflection FTIR measurements are performed for linearly polarized light with 2 cm^−1^ resolution on a Bruker VERTEX 70 v spectrometer coupled to an IR microscope (HYPERION 3000, Bruker). Knife-edge apertures limit the measuring area to 200 μm × 200 μm. Thermal light is incident from the substrate side at a low angle.

Single-color pump-probe spectroscopy is performed using the following setup. A Ti:sapphire regenerative amplifier generates 1 mJ, <100 fs pulses at a 1-kHz repetition rate. The subsequent optical parametric amplifier (2 mm thick β-BaB_2_O_4_, type II) and the difference frequency generator (2-mm-thick AgGaS_2_, type II) produce MIR pulses of 4 μJ energy with a center frequency of 1930 cm^−1^ and a fwhm bandwidth of 280 cm^−1^. A small fraction (~4%) of the MIR pulses is split off with a wedged BaF_2_ window to obtain probe and reference pulses, and the remainder is used for the pump. The pump energy is adjusted with a half-wave plate and a wire-grid polarizer. Down-chirped pump pulses are generated by propagation through a 40-mm-thick CaF_2_ crystal. Pump and probe pulses are focused and overlapped spatially onto the sample by means of an off-axis parabolic mirror with an effective focal length of 4 inch, which are linearly polarized in parallel to the longer axes of the nanoantennas. The incidence angle of the pump is 0°, and that of the probe is <10°. The pump beam with a diameter of 200 μm at the sample spatially covers the probe beam spot of 150 μm diameter. The pump-induced change in absorbance (measured in transmission geometry for the antenna-uncoupled case) and that in reflectance (measured in reflection geometry for the antenna-coupled case) are monitored by the probe pulse with a 2-ps time delay, controlled by a mechanical stage. The spectrum of the transmitted/reflected probe pulse is measured by a monochromator and a MCT 2 × 32 detector array.

## Supplementary information

Supplementary Information

## Data Availability

The data that support the findings of this study are available from the corresponding author upon reasonable request.

## Code availability

The Matlab code of the numerical simulations is available from the corresponding author upon reasonable request.
