# Supplementary material for: Molecular ground-state dissociation in the condensed phase employing plasmonic field enhancement of chirped mid-infrared pulses
Source: Nat Commun. 2019 Aug 29;10:3893. doi: 10.1038/s41467-019-11902-6 (PMC6715752; doi:10.1038/s41467-019-11902-6)
Supplement: Supplementary file 1 — Supplementary Information [file 41467_2019_11902_MOESM1_ESM.pdf]

Supplementary Information

Molecular ground-state dissociation in the condensed phase employing plasmonic field enhancement of chirped mid-infrared pulses

Ikki Morichika, Kei Murata, Atsunori Sakurai, Kazuyuki Ishii, Satoshi Ashihara*

Institute of Industrial Science, The University of Tokyo, 4-6-1, Komaba, Meguro-ku, Tokyo 153-8505, Japan

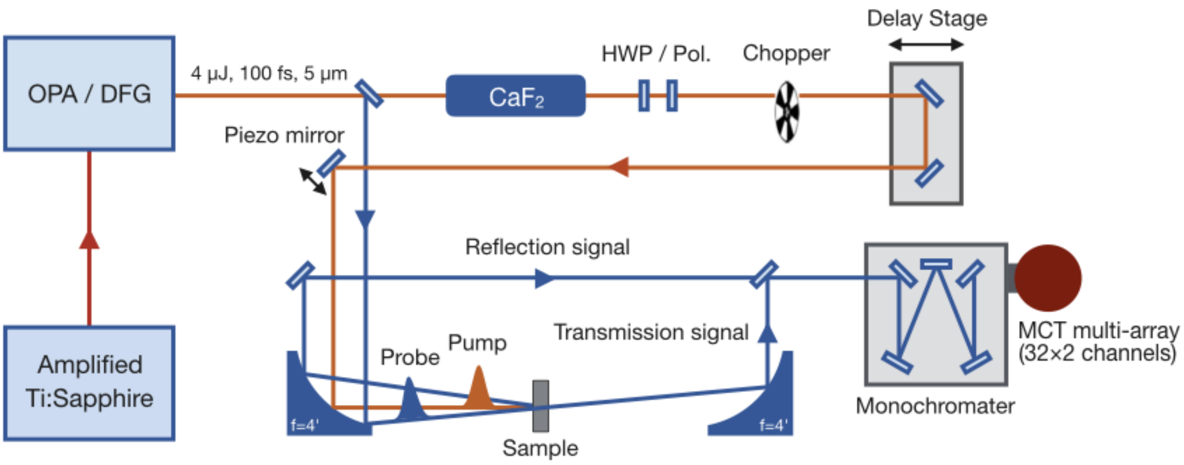

Supplementary Figure 1 | Schematic of the pump-probe spectroscopy system. OPA, DFG, and HWP denote an optical parametric amplifier, a difference frequency generator, and a half-wave plate, respectively. A piezo mirror introduces fast modulation on the pump-probe delay to eliminate a coherent artifact that originates from interference between the probe pulse and the scattered pump pulse.

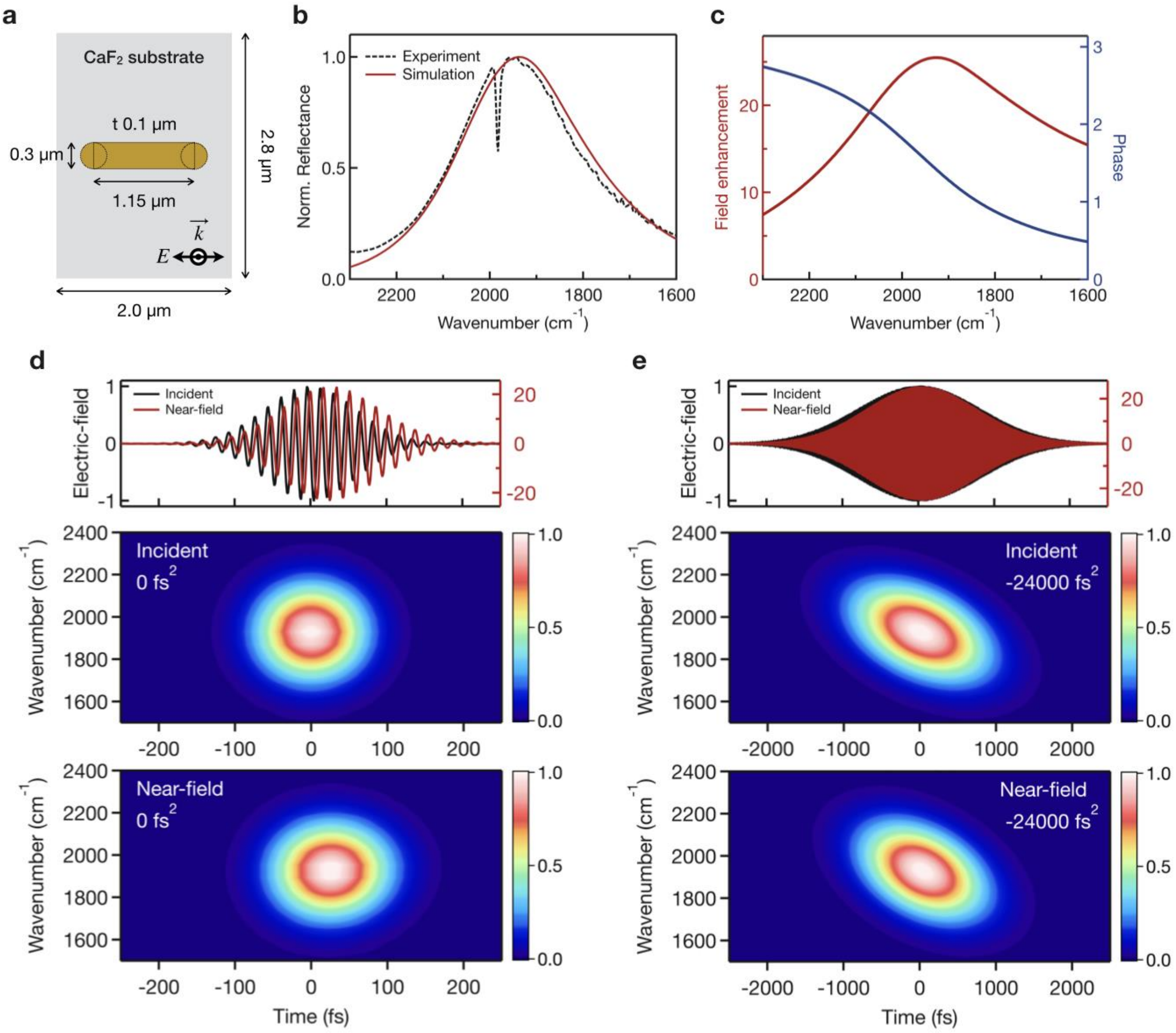

Supplementary Figure 2 | Simulated optical responses of gold nanoantennas. (**a**) Sketch of the geometry used in the FDTD simulations. (**b**) Calculated (red solid) and measured (black dashed) reflectance spectra for the nanoantenna arrays immersed in *n*-hexane solution. (**c**) Simulated electric-field enhancement factor (red) and its phase response (blue). (**d,e**) Calculated temporal waveforms of the incident electric field (black) and the enhanced near-field (red) and the corresponding spectrograms at a distance of 5 nm away from the antenna tip in the direction of the long antenna axis, (d) for the FTL input pulse and (e) for the down-chirped input pulse with GDD = -24,000 fs^2.

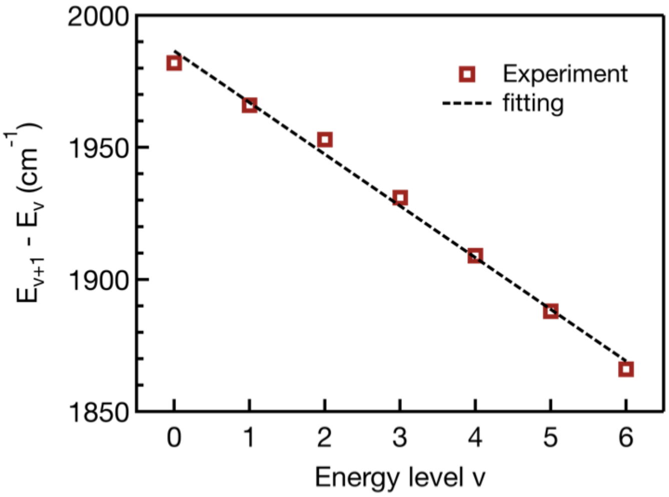

Supplementary Figure 3 | Transition energies of $W(CO)_6$. The resonance frequencies of the ground-state/excited-state absorptions obtained by the pump-probe measurements (markers). The slope of the straight line (black dashed line) corresponds to -2χ_e, where χ_e is the anharmonicity parameter.

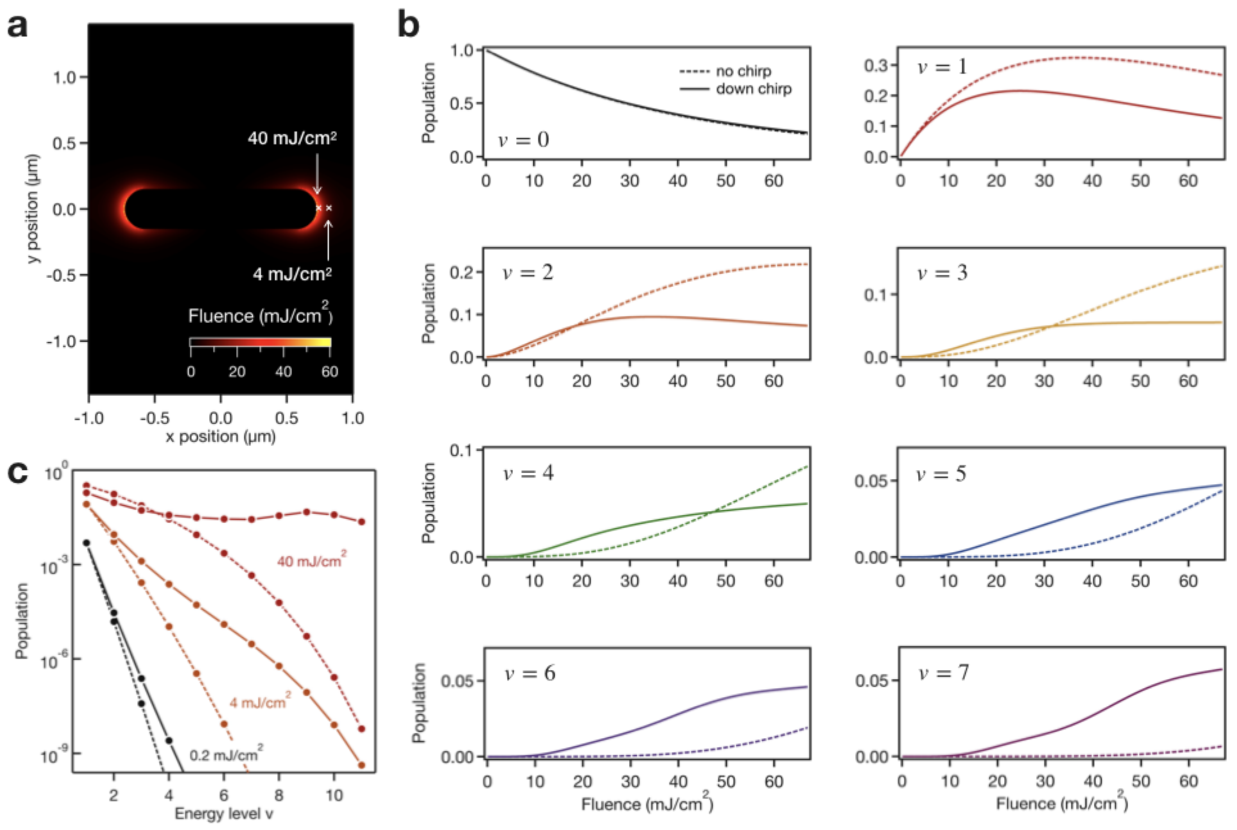

Supplementary Figure 4 | Calculated vibrational population distributions. (**a**) Spatial distribution of optical fluence at z = 0.05 μm (at the middle of the antenna height) created by the pulsed illumination with a fluence of 0.2 mJ cm^{-2}, simulated for a unit cell of 2.0 μm × 2.8 μm. (**b**) Vibrational population for each level as a function of the pump fluence, calculated for the FTL pump pulse (dashed lines) and for the down-chirped pump pulse of GDD = -24,000 fs^2 (solid lines). (**c**) Vibrational population distributions for the pump fluence of 0.2 mJ cm^{-2}, 4 mJ cm^{-2}, and 40 mJ cm^{-2}.

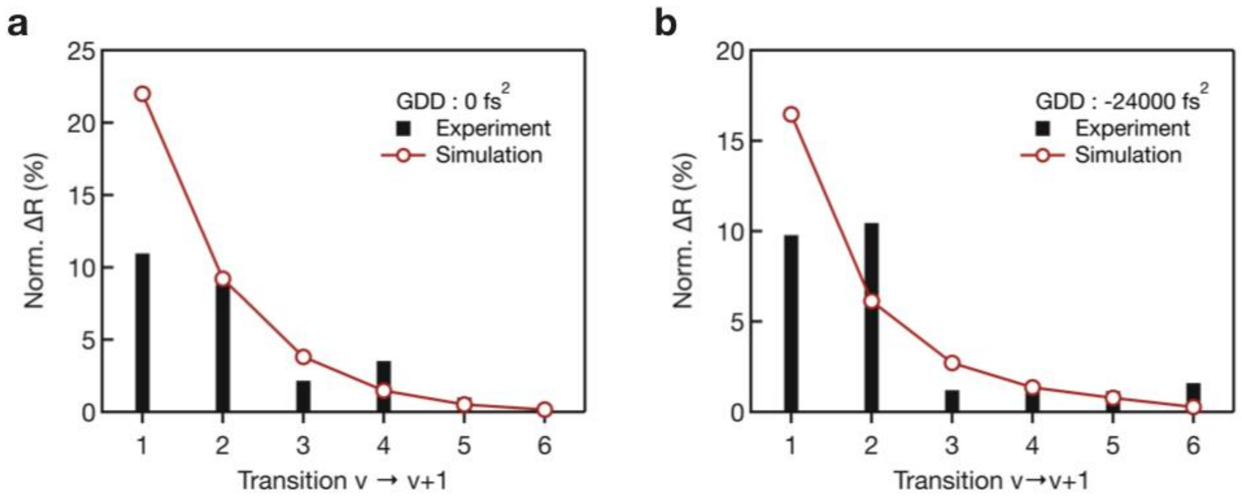

Supplementary Figure 5 | Estimated transient reflectance change. Experimental (black bar) and estimated (red circle) transient reflectance change for (a) the FTL and (b) the down-chirped pump pulses, normalized by the spectral modification of the linear reflectance.

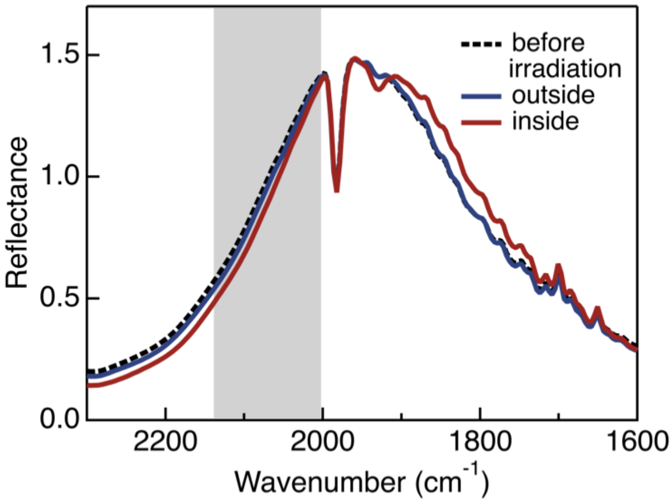

Supplementary Figure 6 | Linear reflectance spectrum before/after an hour of chirped pump irradiation. For any of the three reflectance spectra, absorption dip is not observed in the range of fundamental frequencies for the linear CO adsorbed on gold surfaces (2000–2140 cm^{-1}, gray shaded area): a black dashed line is for the one measured before the irradiation, a blue/red line is for the one measured after the pump irradiation for the area outside/inside the pump beam spot.

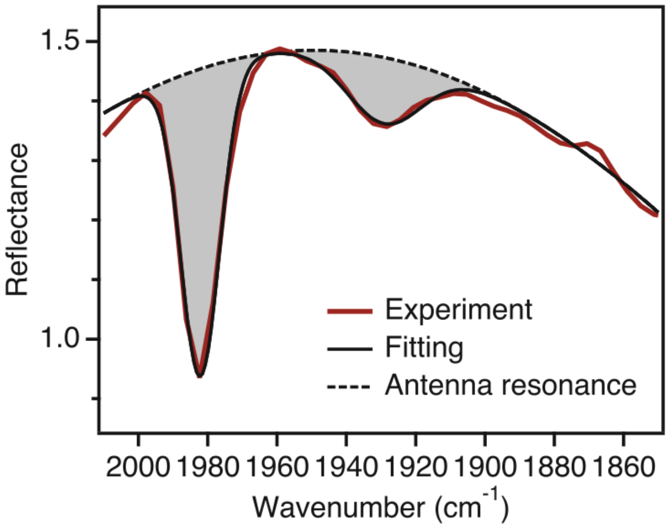

Supplementary Figure 7 | Gaussian-fitting analysis on the linear reflectance spectrum after an hour of chirped pump irradiation. The reflectance spectrum (red line) is fitted with a linear combination of three Gaussian functions (black line): one is for the antenna resonance (black dashed line), another is for the T_{1u} mode of $W(CO)_6$, and the other is for the A_1 mode of adsorbed $W(CO)_5$.

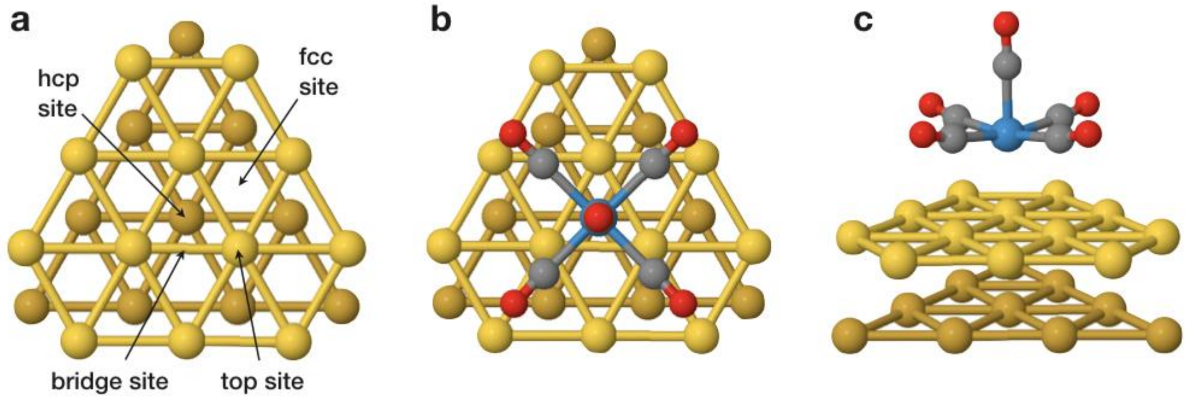

Supplementary Figure 8 | DFT studies of $W(CO)_5$ adsorbed on gold surfaces. (**a**) Au_{22}(12,10) cluster model representing the Au(111) surface with different adsorption sites. (**b,c**) Top (b) and side (c) views of the optimized structures for $W(CO)_5$ adsorbed on the Au(111) surface.

Supplementary Table 1 | DFT calculation results. Binding energies and vibrational frequencies for $W(CO)_5L$ (L = CO, *n*-hexane, H_2O, and none) and $W(CO)_5$ adsorbed on Au(111) surface.

Ligand / Surface	Binding energy ΔG (kcal/mol)	Vibrational frequency ν (cm^{-1})	Frequency shift $\Delta\nu$ (cm^{-1})
**CO**	-32.1	T_{1u} 2004	-
***n*-hexane**	-0.5	A_1 1961	-43
**H_2O**	-15.7	A_1 1953	-51
**Au(111) hcp site**	-4.9	A_1 1959	-45
**none**		A_1 1958	-46

Supplementary Note 1: Spectral/temporal properties of plasmonic near-fields.

To investigate the spectral/temporal properties of plasmonic near-fields, finite-difference time-domain (FDTD) simulations are performed. As shown in Supp. Fig. 2a, each nanoantenna is modeled using a rectangle (1.15 μm long, 0.3 μm wide, and 0.1 μm high) and two half-cylinders (0.3 μm in diameter and 0.1 μm high) at both ends. The antenna is placed on a CaF_2 substrate with a refractive index of 1.41 and immersed in a *n*-hexane solution with an index of 1.375. A mesh size around the antenna is set to 5 nm. All simulations are performed under periodic boundary conditions in the *xy*-direction (2.0 μm × 2.8 μm), and under perfectly-matched boundary condition in the *z*-direction. The near-field coupling among the antennas along the *x*-axis is negligible. The arrangement period of 2.8 μm approximately satisfies the critical coupling condition[1] for the fundamental frequency of the T_{1u} mode of $W(CO)_6$. The simulated reflectance spectrum shown in Supp. Fig. 2b is in good agreement with the measured spectrum, ensuring the validity of our simulations.

Supplementary Figure 2c shows the amplitude and the phase of the near-field enhancement at a distance of 5 nm away from the antenna tip in the direction of the long antenna axis. At around the plasmon resonance, the second-order dispersion is negligibly small (because of the nature of the inflection point). Therefore, the third- and higher-order dispersions may modify the temporal waveform of the enhanced near-field. Supplementary Figure 2d shows the calculated waveform and its spectrogram of the enhanced near-field for a Fourier-transform-limited (FTL) incident pulse with a center frequency of 1930 cm^{-1}, a temporal duration of 100 fs, and linear polarization along the long antenna axis. The peak electric-field is enhanced by 23 times and the temporal duration is slightly elongated from 100 fs to 110 fs. Supplementary Figure 2e shows the calculated waveform and its spectrogram of the enhanced near-field upon illumination of a down-chirped pulse with group-delay dispersion (GDD) of -24000 fs^2. The peak electric-field is enhanced by 25 times, and the temporal duration is slightly compressed from 1.3 ps to 1.2 ps. These results indicate that the substantially-enhanced mid-IR pulsed fields are generated in the vicinity of the antenna, with almost the same temporal waveforms (except for the $\pi/2$ phase shift in the carrier oscillation) as the incident ones for both the FTL and the down-chirped pulses.

Supplementary Note 2: Numerical calculations of vibrational populations.

In our pump-probe measurements for the antenna-coupled molecules, molecular vibration is excited by plasmonic near-fields of pump pulses. Because the plasmonic near-field is spatially confined on a nanometer scale as shown in Supp. Fig. 4a, the vibrational populations should vary considerably in space, i.e., the molecular vibration is excited more efficiently at the hot spots.

To investigate the pump-fluence dependence of the multi-quantum vibrational excitations, we numerically calculate the time evolution of the density matrix $\rho(t)$ by solving the following equations with the fourth-order Runge–Kutta method[2,3].

$$\frac{\partial}{\partial t}\rho_{nm} = -\frac{i}{\hbar}[H,\rho]_{nm} - \gamma_{nm}\rho_{nm}, \tag{1}$$

$$\frac{\partial}{\partial t}\rho_{nn} = -\frac{i}{\hbar}[H,\rho]_{nn} + \Gamma_m\rho_{mm} - \Gamma_n\rho_{nn} \qquad (m > n). \tag{2}$$

Here, γ_{nm} and Γ_n (m, n: non-negative integers) represent the coherence relaxation rate and the population relaxation rate, respectively. The excitation pulse is modeled as a Gaussian pulse (with a center frequency of 1930 cm^{-1} and a temporal duration of 100 fs in FTL) without/with linear down-chirp of GDD = -24000 fs^2. The T_{1u} CO-stretch in tungsten hexacarbonyl is modeled as a one-dimensional Morse oscillator, where the anharmonic vibrational ladder comprises 12 energy levels with E_v's ($v = 0, 1, 2, \cdots 11$) expressed by $E_v = 2007 \cdot v - 10 \cdot v(v+1)$ [cm^{-1}] (deduced from the transition energies obtained by the pump-probe measurements, see Supp. Fig. 3). Considering the small anharmonicity of $\chi_e \approx 0.0049$, we assume that the transition dipole moment μ follows the harmonic-oscillator formula: $\mu_{v,v+1} = \sqrt{v+1} \cdot \mu_{0,1}$. The population lifetime of 150 ps for $v = 1$, and that decreasing by a factor of two for each ascending level[4] are used in the calculations. The relaxation rate of coherence between $v = n$ and $v = n+1$ states is assumed to be $0.2 \times (n+1)$ ps^{-1} to be consistent with the experimentally observed linewidths. In fact, there are other relaxation mechanisms (rotational diffusion, intramolecular energy transfer among the three degenerate T_{1u} modes[5], and vibrational energy transfer into E_g CO-stretching mode[6], etc.), which may reduce excitation efficiencies. Here we use an "effective" transition dipole moment $\mu_{0,1} =$ 0.35 Debye (3 times smaller than the previously reported values[7]) in our series of numerical simulations so that the measured transient absorption of the uncoupled system shown in Fig. 3a in the main manuscript is quantitatively reproduced.

The calculated vibrational population for each level ($v = 0 - 7$), evaluated at the pump-probe time delay of 2 ps, is plotted as a function of the pump fluence in Supp. Fig. 4b. The population distributions for three representative pump fluences of 0.2 mJ cm^{-2} (without plasmonic enhancements), 4 mJ cm^{-2} (160 nm away from the antenna tip), and 40 mJ cm^{-2} (5 nm away from the tip) are also displayed in Supp. Fig. 4c. As expected, the vibrational ladder is climbed more efficiently for higher fluence, i.e., at the plasmonic hot spots. In this calculation, we reduce the field enhancement factor by $\sqrt{3}$ compared with the one obtained from FDTD simulations (shown in Supp. Fig. 2c) so that the measured transient reflection of antenna-coupled system is well-reproduced (Supp. Note 3 and Supp. Fig. 5). Such degradation in the field enhancement may arise from non-ideal antenna shape and unwanted contamination at gold surfaces in our experiments.

In general, there is an optimal amount of chirp for efficient excitation into higher-lying states, which depends on the coherence relaxation rate and the pump fluence. In fact, our series of simulations indicate that down-chirp of GDD = -24000 fs^2 is less than the optimal amount. This down-chirp, however, certainly contributes in raising the vibrational population to higher-lying states for all of the three fluences, as can be seen in Supp. Fig. 4c.

Supplementary Note 3: Numerical calculations of transient reflectance change.

In the same manner as the pump process, our antenna-enhanced pump-probe spectroscopy preferentially probes the molecules that exist in the plasmonic hot spots. Here, we calculate the transient reflectance change by incorporating the above-mentioned numerical simulations into a coupled point-dipole model[8].

In this model, we consider two point dipoles: one represents a plasmon mode of an antenna, and the other represents a vibrational mode of a molecule. They are characterized by the complex polarizabilities $\tilde{\alpha}_{\mathrm{ant}}(\omega)$ and $\tilde{\alpha}_{\mathrm{vib}}(\omega)$, respectively. For simplicity, we use a scalar description. Now we consider the case where the driving frequency ω_0 is in resonance with both of the antenna and the molecular vibration. In this condition, the complex polarizabilities can be regarded as pure imaginary, expressed as, $\tilde{\alpha}_{\mathrm{ant}}(\omega_0) = i\alpha_{\mathrm{ant}}$, $\tilde{\alpha}_{\mathrm{vib}}(\omega_0) = i\alpha_{\mathrm{vib}}$.

The incident electric field $\tilde{E}_{\mathrm{in}}$ (polarized along the long antenna axis) excites a plasmon and produces a near-field. This near-field in turn induces a vibrational dipole:

$$\tilde{p}_{\mathrm{vib}} = i\alpha_{\mathrm{vib}} \cdot G(\mathrm{r}_{\mathrm{vib}}, \mathrm{r}_{\mathrm{ant}}) \cdot i\alpha_{\mathrm{ant}} \cdot \tilde{E}_{\mathrm{in}}. \tag{3}$$

Here $\mathrm{r}_{\mathrm{ant}}$ and $\mathrm{r}_{\mathrm{vib}}$ denote positions of the antenna and the molecule, respectively. $G(\mathrm{r}, \mathrm{r}_0)$ is the Green function describing an electric field at r produced by a radiating unit dipole located at r_0. In this expression, the direct contribution of the incident field onto the vibrational polarization is neglected since it is much smaller than that induced by the enhanced near-field. Equation 3 indicates that the antenna, in effect, amplifies the vibrational polarization by $\alpha_{\mathrm{ant}}G$, which corresponds to the field enhancement factor f. The induced vibrational dipole acts back to the antenna, by producing additional antenna polarization:

$$\Delta\tilde{p}_{\mathrm{ant}} = i\alpha_{\mathrm{ant}} \cdot G(\mathrm{r}_{\mathrm{ant}}, \mathrm{r}_{\mathrm{vib}}) \cdot \tilde{p}_{\mathrm{vib}}. \tag{4}$$

From these expressions, we obtain the effective dipole moment $\tilde{p}_{\mathrm{ant,eff}}$ and the effective polarizability of the antenna $\alpha_{\mathrm{ant,eff}}$ as

$$\tilde{p}_{\mathrm{ant,eff}} = i(\alpha_{\mathrm{ant}} - (\alpha_{\mathrm{ant}}G)^2\alpha_{\mathrm{vib}})\tilde{E}_{\mathrm{in}} \equiv i\alpha_{\mathrm{ant,eff}} \cdot \tilde{E}_{\mathrm{in}}. \tag{5}$$

By considering that the reflectance of the antenna arrays is proportional to the square of the antenna polarizability, we arrive at the following expression for the modification of the reflectance due to the coupling with the molecule:

$$\Delta R \propto |\alpha_{\mathrm{ant}}|^2 - |\alpha_{\mathrm{ant,eff}}|^2 \cong \alpha_{\mathrm{ant}}(\alpha_{\mathrm{ant}}G)^2\alpha_{\mathrm{vib}}. \quad (6)$$

Here, we assume that the intrinsic polarizability of the antenna to be much larger than the modification. This equation indicates that the spectral modification observed in the reflection spectra originates from the absorption of the molecule, enhanced by the square of the field enhancement factor $(\alpha_{\mathrm{ant}}G)^2 = f^2$.

Because Eq. 6 is linear in α_{vib}, it can be generalized into a case where an antenna is coupled with multiple molecules:

$$\Delta R \propto \frac{N}{V}\int_V (f(\mathrm{r}_{\mathrm{vib}}))^2\,\alpha_{\mathrm{vib}}dV. \quad (7)$$

Here, N represents the number density of the molecules, and V represents the volume where the molecules are present.

In pump-probe measurements, the *n*-th excited-state absorption is proportional to $|\mu_{n,n+1}|^2 \cdot (P_n - P_{n+1})$, where P_n is the population of $v = n$. By plugging this expression into Eq. 7, we obtain the following expression for the transient reflectance change attributed to the *n*-th excited-state absorption:

$$\Delta R_{n,n+1} \propto \frac{N}{V}\int_V (f(\mathrm{r}_{\mathrm{vib}}))^2 \cdot \left(P_n(\mathrm{r}_{\mathrm{vib}}) - P_{n+1}(\mathrm{r}_{\mathrm{vib}})\right) \cdot |\mu_{n,n+1}|^2 dV. \quad (8)$$

This formula is viewed as a spatially-averaged excited-state absorption signal, weighted with the near-field intensity enhancement factor.

We now calculate the transient reflectance change using Eq. 8. Here, the field enhancement factor $f(\mathrm{r}_{\mathrm{vib}})$ and the population distribution $P_n(\mathrm{r}_{\mathrm{vib}})$ are obtained from the FDTD simulations (Supp. Fig. 4a) and the numerical calculations of the time evolution of the density matrix (Supp. Fig. 4b), respectively. The spatial integration is taken for the plane within the unit cell (2.0 μm × 2.8 μm at z = 0.05 μm). In Supp. Fig. 5, we present the transient reflectance changes for the experiments (bars) and for the calculations (markers), normalized by the spectral modification of the linear reflectance (Eq. 7). As can be seen, we realize a reasonable agreement between them.

Supplementary Note 4: Hot-spot volume in the vicinity of the nanoantenna tip.

In our antenna-enhanced pump-probe measurements, the only molecules located within the plasmonic hot spots are efficiently excited and sensitively detected (Supp. Notes 2 and 3). Because the diffusion distance of small molecules per 2 ms (our pump-pulse interval) is typically in the order of micrometers, the species dissociated with a pump pulse would diffuse out of the hot spot before the next pump-pulse comes. This indicates that the free CO, $W(CO)_5$(*n*-hexane), and $W(CO)_5(H_2O)$ are undetectable in our antenna-enhanced pump-probe measurements.

Regarding the original species of $W(CO)_6$, fresh molecules are continuously supplied into the hot spot because the volume of the whole bulk solution within the sample cell is much larger (by ~10^9 times) than the total hot-spot volume within the irradiation beam area. Therefore, it is reasonable that no depletion of the absorption dip of the T_{1u} mode is observed in the steady-state reflectance shown in Supp. Fig. 6.

Supplementary Note 5: Estimation of reaction efficiency.

As shown in Eq. 7, the spectral modification observed in the reflection spectra is proportional to the number of molecules and the transition dipole moment of the molecular vibration. From theoretical calculations based on density functional theory (see Supp. Note 6), the transition dipole moment for A_1 mode of adsorbed $W(CO)_5$ is found to be half of that for T_{1u} mode of $W(CO)_6$. By Gaussian-fitting analysis on the reflectance spectrum (Supp. Fig. 7), the number ratio of adsorbed $W(CO)_5$ to dissolved $W(CO)_6$ in a hot spot is found to be 0.84 after the 1-h irradiation. Considering also the pump-pulse interval of 2 ms, we estimate the 'single-shot' reaction efficiency, which is the number ratio of adsorbed $W(CO)_5$ produced per pump pulse to dissolved $W(CO)_6$ in a hot spot, to be ~10^{-9}.

Supplementary Note 6: DFT studies of $W(CO)_5$ adsorption on gold surfaces.

To investigate the binding energies and vibrational frequencies of the decarbonylated species $W(CO)_5$ adsorbed on the gold surface, theoretical calculations based on density functional theory (DFT) are performed using the M06 functional as implemented in the Gaussian 16 package. A basis set composed of SDD for tungsten and gold atoms and 6-311G(d,p) for other atoms is used for structural optimizations and frequency calculations. The gold cluster model of Au_{22}(12,10) (i.e. 12 atoms in the first and second layers) shown in Supp. Fig. 8a is chosen to represent the Au(111) surface. The gold surfaces are constructed using the bulk lattice constant of 4.078 Å.

In general, there are four different adsorption sites on the gold surfaces: top site, bridge site, fcc (face centered cubic) site, and hcp (hexagonal close packed) site (Supp. Fig. 8a). In the present calculations, W atom of $W(CO)_5$ is placed on each of the different sites and the geometry optimization of $W(CO)_5$ above the surface is carried out while the geometry of the metal cluster is fixed. The hcp site is found to be most stable on the Au(111) surface: the optimized structure is shown in Supp. Figs. 8b and 8c.

The Gibbs free binding energy (ΔG) is calculated according to the formula

$$\Delta G = G(\mathrm{W(CO)_5L}) - G(\mathrm{W(CO)_5}) - G(\mathrm{L}), \qquad (9)$$

where G(X) denote the calculated Gibbs free energies of X compounds. For comparison, the binding energies of $W(CO)_5L$ (L = CO, *n*-hexane, and H_2O) are also calculated. The calculated binding energies are summarized in Supp. Table 1. It is apparent that the decarbonylate species can certainly be adsorbed on the gold surface. The binding energy of $W(CO)_5$ on the gold surface is larger than that of $W(CO)_5$(*n*-hexane) while smaller than that of $W(CO)_5(H_2O)$. This fact suggests that some of the decarbonylate species may be combined with a water molecule (a little impurity in the solvent). The species $W(CO)_5(H_2O)$, however, would not be detectable in our antenna-enhanced reflection measurements as discussed in Supp. Note 4.

The calculated vibrational frequencies of the T_{1u} mode of $W(CO)_6$ and the A_1 mode of $W(CO)_5L$ are summarized in Supp. Table 1. The frequency of A_1 CO-stretching mode of $W(CO)_5$ adsorbed on the gold surface is red-shifted by 45 cm^{-1} compared with that of the T_{1u} mode of $W(CO)_6$. This frequency shift is close to 53cm^{-1}, the one observed in our experiments. Note that the calculated frequency shifts for L = *n*-hexane, H_2O are in reasonable agreement with previously reports (55 cm^{-1} for L = H_2O, 57 cm^{-1} for L = CH_3CN, and 51 cm^{-1} for L = Ar, Kr, Xe[5,9,10]).

The red-shift of the A_1 mode frequency in $W(CO)_5$-Au(111) is mainly attributed to the loss of CO ligand in $W(CO)_6$. This is because the CO dissociation strengthens the back-donation of the W center to the other CO ligand located at the opposite side of the dissociated site and weakens the corresponding C≡O bond. On the other hand, the effect on the adsorption of the dissociated product to the Au surface is considered to be rather small though both the CO ligands and W center slightly interacts with the Au surface to stabilize the coordinatively unsaturated site. Indeed, the theoretical results based on DFT calculation well agree with this interpretation: the calculated frequency of the A_1 mode in $W(CO)_5$-Au(111) (1959 cm^{-1}) is significantly shifted to the red-side compared to that of the T_{1u} mode in $W(CO)_6$ (2004 cm^{-1}) while it is similar to that of the A_1 mode in $W(CO)_5$ (1958 cm^{-1}).

Supplementary References

1. Adato, R. et al. Ultra-sensitive vibrational spectroscopy of protein monolayers with plasmonic nanoantenna arrays. *Proc. Natl. Acad. Sci. U.S.A.* **106,** 19227–19232 (2009).
2. Witte, T. et al. Controlling molecular ground-state dissociation by optimizing vibrational ladder climbing. *J. Chem. Phys.* **118,** 2021–2024 (2003).
3. Witte, T., Yeston, J. S., Motzkus, M., Heilweil, E. J. & Kompa, K. L. Femtosecond infrared coherent excitation of liquid phase vibrational population distributions (v>5). *Chem Phys Lett* **392,** 156–161 (2003).
4. Strasfeld, D. B., Shim, S.-H. & Zanni, M. T. Controlling vibrational excitation with shaped mid-IR pulses. *Phys. Rev. Lett.* **99,** 038102 (2007).
5. Banno, M., Iwata, K. & Hamaguchi, H. Intra- and intermolecular vibrational energy transfer in tungsten carbonyl complexes $W(CO)_5(X)$ (X=CO, CS, CH_3CN, and CD_3CN). *J. Chem. Phys.* **126,** 204501–204501 (2007).
6. Tokmakoff, A., Sauter, B., Kwok, A. S. & Fayer, M. D. Phonon-induced scattering between vibrations and multiphoton vibrational up-pumping in liquid solution. *Chem. Phys. Lett.* **221,** 412–418 (1994).
7. Melnikoff, A. & Beck, W. Absolute Infrared intensities and atomic polarizations of ‘symmetric’ (dipole moment free) metal carbonyls $M(CO)_6$ (M = Cr, Mo, W), $Fe(CO)_5$, $M_2(CO)_{10}$ (M = Mn, Re). *Z. anorg. allg. Chem.* **641,** 606–609 (2014).
8. Rezus, Y. L. A. & Selig, O. Impact of local-field effects on the plasmonic enhancement of vibrational signals by infrared nanoantennas. *Opt. Express* **24,** 12202–12227 (2016).
9. Greetham, G. M. et al. Time-resolved multiple probe spectroscopy. *Rev. Sci. Instrum.* **83,** 103107 (2012).
10. Sun, X.-Z., George, M. W., Kazarian, S. G., Nikiforov, S. M. & Poliakoff, M. Can organometallic noble gas compounds be observed in solution at room temperature? A time-resolved infrared (TRIR) and UV spectroscopic study of the photochemistry of $M(CO)_6$ (M = Cr, Mo, and W) in supercritical noble gas and CO_2 solution. *J. Am. Chem. Soc.* **118,** 10525–10532 (1996).
